# COVID-19 in Latin America: Novel transmission dynamics for a global pandemic?

**DOI:** 10.1371/journal.pntd.0008265

**Published:** 2020-05-07

**Authors:** Matthew J. Miller, Jose R. Loaiza, Anshule Takyar, Robert H. Gilman

**Affiliations:** 1 University of Oklahoma, Norman, Oklahoma, United States of America; 2 University of Alaska Fairbanks, Fairbanks, Alaska, United States of America; 3 INDICASAT-AIP, City of Knowledge, Republic of Panama; 4 Smithsonian Tropical Research Institute, Balboa, Republic of Panama; 5 John Hopkins University, Baltimore, Maryland, United States of America; London School of Hygiene & Tropical Medicine, UNITED KINGDOM

The COVID-19 virus expanded from China into Western Asia, Europe, and North America, impacting many of the world’s wealthiest countries. Brazil reported Latin America’s first case in late February 2020, and in less than a month, over 7,000 COVID-19 cases have been confirmed among nearly every country and territory in Latin America and the Caribbean (LAC). The LAC outbreak appears to be about two weeks behind the United States and Canada and about three to four weeks behind Western Europe. Thus, the global COVID-19 pandemic is entering a new phase, not only expanding beyond primarily temperate Northern Hemisphere countries into the tropics but also spreading to a geopolitical region marked by significantly worse poverty, water access and sanitation, and distrust in public governance (Fig 1). We believe that these aspects of the Latin American context are likely to substantially affect the transmission dynamics and scope of the COVID-19 outbreak in LAC, with potential implications for the trajectory of the global pandemic.

**Fig 1:**
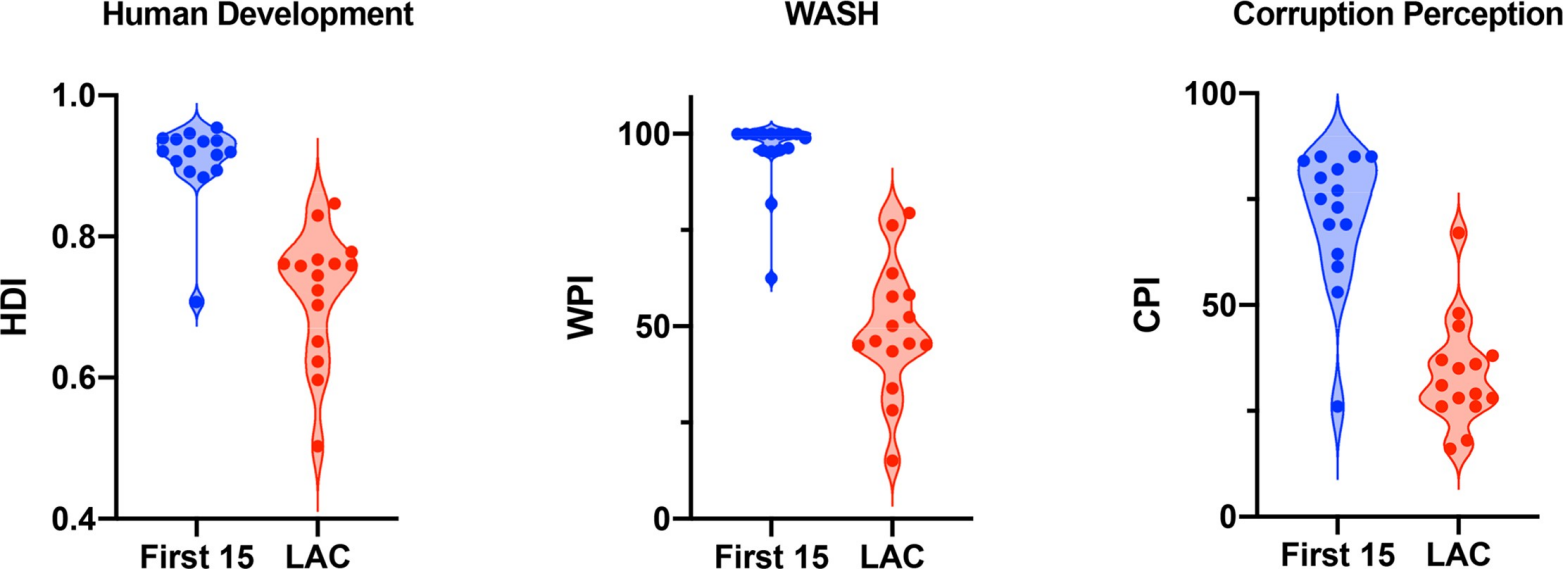
Socioeconomic differences between “First 15” COVID-19 countries and LAC. Significant differences are found in the HDI [23], WPI [24], and CPI [25] between the first 15 (“First 15”) countries where COVID-19 was recorded to have expanded rapidly out of China (blue) and the 15 most populous countries in LAC (red). HDI: (Welch-corrected *t* test; Average_First 15_ = 0.907; Average_LAC_ = 0.721; *P* < 0.0001); WASH: (Welch-corrected *t*-test; Average_First 15_ = 95.11; Average_LAC_ = 49.17; *P* < 0.0001); CPI: (Welch-corrected *t*-test, Average_First 15_ = 70.9; Average_LAC_ = 33.87; *P* < 0.0001). We classified “First 15” countries as the 15 non-Chinese countries with the highest reported number of COVID-19 cases in the March 8, 2020 COVID-19 Situation Report [26]. CPI, Corruption Perceptions Index; HDI, Human Development Index; LAC, Latin America and the Caribbean; WASH, Water, Sanitation, and Hygiene; WPI, Water Poverty Index.

## COVID-19, temperature and humidity, and transmission

One of the most important questions in COVID-19 global epidemiology is whether warmer temperature and higher humidity impedes transmission. The initial countries to experience the largest increase in day over day new COVID-19 cases experienced cold and dry conditions typical for wintertime in temperate Northern Hemisphere. Among Chinese cities, the COVID-19 basic reproductive number (*R*) appears to be inversely related with temperature and relative humidity, albeit with substantial variation [1]. One early travel-based model of COVID-19 global spread predicted that several southeastern Asian countries should have been the first non-Chinese countries to experience substantial COVID-19 outbreaks [2]. Instead, substantial outbreaks outside China occurred first in Western Asia and Europe. Additional support for the hypothesis that higher temperature and humidity dampens COVID-19 transmission comes from laboratory experiments on the severe acute respiratory syndrome (SARS) virus and other coronaviruses, which found that increasing temperature and humidity decreases the virulence of dried virus on smooth surfaces [3].

Some commentators have suggested that COVID-19 transmission may decline as the Northern Hemisphere transitions to summer, as happens with seasonal influenza. However, as demonstrated by 2009 H1N1 influenza, novel pandemic respiratory virus transmission dynamics are often decoupled from the climatic conditions that drive the seasonality of influenza [4]. While seasonal influenza does vary with temperature and humidity in LAC, the region’s environmental heterogeneity causes peaks in influenza transmission to be asynchronous across the region [5]. Thus, although environmental conditions in March 2020 appear to be less favorable for COVID-19 transmission across most of LAC, by July 2020 many South American cities have climatic conditions that would appear more favorable for rapid COVID-19 transmission [1], coinciding with a strong peak of seasonal influenza transmission in subtropical South America between May and October [6]. Therefore, while the environmental models suggest that LAC’s higher temperature and humidity may slow the initial COVID-19 transmission, this effect may be ephemeral for much of the region. Any tropical climate effect may also be limited by the ubiquity of indoor air conditioning, which creates indoor environments with temperature and humidity ranges favorable to coronavirus persistence [3]. Most importantly, climate-based transmission models assume COVID-19 spreads primarily via indirect surface contact transmission. We believe that other transmission models (especially fecal–oral) may be as or more important for COVID-19 transmission in LAC, making predictions from climate models premature.

## The potential for increased fecal–oral COVID-19 transmission in LAC

Although a respiratory disease, COVID-19 is likely transmissible via fecal–oral contamination. While only a portion of Wuhan patients experienced gastrointestinal symptoms, these generally presented prior to respiratory symptoms [7]. Fecal swabs test positive using reverse transcription PCR (RT-PCR) for COVID-19 virus in slightly more than half of sampled patients [8], and stool samples remained positive for an average of 11 days after respiratory swabs turned negative [8]. During the Middle East respiratory syndrome (MERS) and SARS (and now COVID-19) coronavirus epidemics, patients often experienced gastrointestinal symptoms, and these viruses were detected in stool samples and shown to infect and replicate in intestinal tissues [9]. A large SARS outbreak in a Hong Kong apartment complex is believed to be due to virus particles that were aerosolized from improperly installed wastewater pipes [10]. Finally, molecular modeling suggests that the COVID-19 (like MERS and SARS) uses the angiotensin-converting enzyme II (ACE2), which is highly expressed in both lung and some intestinal epithelial tissues [11] as its host receptor. Collectively, this suggests that fecal–oral transmission will probably be important for COVID-19 spread [9].

Thus, LAC will be the first region where water scarcity and poor sanitation may substantially impact COVID-19 spread. The World Bank estimates that 36 million people in LAC lack access to improved drinking water, and 110 million lack access to improved sanitation [12]. In LAC urban slums, the lack of in-house water delivery results in reduced water usage, limited handwashing, and poor family hygiene, leading to widespread fecal contamination [13]. In LAC households without clean water delivery, drinking water is often boiled and stored; yet this water often becomes fecally contaminated [13]. Importantly, coronaviruses can remain infectious for weeks in room temperature water [14]. Like poor clean water access, inadequate sewage disposal causes chronic fecal contamination and disease in LAC, even when improved water is available [15].

Many LAC countries score poorly on the WASH index, which is a measure of access to abundant clean water and improved sanitation. If increased transmission due to fecal contamination is combined with climatically reduced contact transmission, the epidemiological dynamics of COVID-19 in LAC may be fundamentally distinct from the dynamics currently observed in the Northern Hemisphere. We can look to the epidemiological characteristics of norovirus and cholera in LAC for insights. In LAC slums with poor water access and sanitation, over 80% of children are infected with at least one strain of norovirus in their first year of life [16]; adults are only infected when novel genotypes enter the community. Cholera is a disease of poverty exacerbated by poor access to clean water. During the 1991 cholera epidemic in Peru, cholera spread nearby instantaneously from a single town to nearly communities along the Peruvian coast with attack rates over 2% in just the first month of the epidemic [17]. Because cholera is often transmitted via contaminated stored water and food, up to half of all family members show signs of infection within two days of the presentation of an index case [18]. If COVID-19 spreads in a similar fashion, we can expect increased intrafamily and intraneighborhood infection rates. Like norovirus, this may result in rapid herd immunity within infected communities [16]; however, with a large peak of simultaneous infections, local health centers will almost certainly be overwhelmed. Extreme rates of local infection can cause complex metapopulation dynamics that could favor rapid local eradication while at the same time facilitating long-term regional viral persistence [19]. In the face of this, LAC will need to implement widespread population surveillance of both active cases (using RT-PCR) and prior exposure and potential immunity via serology.

## COVID-19, weak infrastructure, and poverty

COVID-19 expanded from China into some of the world’s richest countries (Fig 1), perhaps masking socio-economic factors in the outbreak’s spread. During recent epidemics, LAC’s poor were more likely to become infected with Zika and more likely bear children with microcephaly [20], suggesting that the burden of COVID-19 may be disproportionately borne by LAC’s poorest and most marginalized. Health infrastructure is weak and inadequate in LAC, where epidemics routinely overwhelm a public health system that suffers from chronic understaffing and a lack of modern medical equipment and diagnostic and therapeutic consumables, including personal protective equipment. If the COVID-19 epidemic in LAC is severe, it is probable that the region will come out of the epidemic even more inequitable than it is now.

Thus, the imperative to “flatten the curve” is even greater for LAC than Western Europe and the United States. Not surprisingly, several LAC countries rapidly implemented strict social restrictions (“lockdowns”) to curb transmission, including complete border closures, restricted daytime movements, night-time curfews, and the cessation of intraprovincial travel. Evidence from China suggests that such extreme restrictions should reduce transmission and blunt COVID epidemics. But will LAC citizens comply? Public distrust of government is significantly higher in LAC than in the first countries to experience COVID-19 spread out of China (Fig 1), and this distrust has been shown to erode compliance with public health societal restrictions [21]. Collectively, the interactions between climate, WASH conditions, and other socioeconomic factors suggest that the impacts of COVID-19 in LAC will be more extreme than even that experienced by Western Europe and the United States. Experimental studies and modeling efforts should focus on alternative COVID-19 transmission dynamics, and LAC’s leaders must continue to take immediate and decisive actions to slow the spread of COVID-19. Extreme regulation of social distancing may be required. Fortunately, several commercial ELISA tests predict neutralizing antibody levels for COVID-19 [22]. Widespread serological testing will allow citizens with developed immunity to return back into society and the economy.
